# Ni-Co bimetal decorated carbon nanotube aerogel as an efficient anode catalyst in urea fuel cells

**DOI:** 10.1038/s41598-018-37011-w

**Published:** 2019-01-24

**Authors:** Robel Mehari Tesfaye, Gautam Das, Bang Ju Park, Jihyeon Kim, Hyon Hee Yoon

**Affiliations:** 1 Department of Chemical and Biological Engineering, Gachon University, Seongnam, Gyeonggi-do 13120 Republic of Korea; 2 Department of Electronic Engineering, Gachon University, Seongnam, Gyeonggi-do 13120 Republic of Korea

## Abstract

Ni-based catalysts have been considered as an efficient anode material for urea fuel cells due to the low cost and high activity in alkaline media. Herein, we demonstrate that Ni-Co bimetallic nanoparticles decorated carbon nanotube aerogels as catalysts for urea oxidation reaction (UOR) can be synthesized by a polyol reduction and sol-gel method. The morphology, structure, and composition of the Ni-Co/MWCNT aerogels were characterized by scanning electron microscopy and X-Ray diffraction. The electro-catalytic activity of the Ni-Co/MWCNT aerogels towards UOR was investigated using cyclic voltammetry. It was found that the Co-doping at 25% (Co/Ni) significantly increased the oxidation peak current and reduced the overpotential of the UOR. Furthermore, the MWCNT aerogel support also remarkably enhanced electro-catalytic activity by providing a high surface area and fast mass transport for the UOR owing to the porous 3D network structures with uniform distribution of Ni-Co nanoparticles. Urea/O_2_ fuel cell with Ni-Co/MWCNT aerogel as anode material exhibited an excellent performance with maximum power density of 17.5 mWcm^−2^ with an open circuit voltage of 0.9 V. Thus, this work showed that the highly porous three-dimensional Ni-Co/MWCNT aerogel catalysts can be used for urea oxidation and as an efficient anode material for urea fuel cells.

## Introduction

Urea fuel cell (UFC) has received considerable attention in recent years owing to the affordability, easy storage/transportation, non-toxic, and non-flammable properties of urea^1,2^. Furthermore, urine and urea-containing wastes can be used for UFCs as fuels, generating electricity and denitrifying waste water simultaneously^3,4^. The UFC is operated as an anion exchange membrane (AEM) fuel cell. The reactions in UFCs are as follows^5^:

1 $${\rm{Anode}},{\rm{CO}}{({{\rm{NH}}}_{2})}_{2}+6{{\rm{OH}}}^{\mbox{--}}\to {{\rm{N}}}_{2}+{{\rm{CO}}}_{2}+5{{\rm{H}}}_{2}{\rm{O}}+6{{\rm{e}}}^{\mbox{--}},E{^\circ }=\mbox{--}0.746\,{\rm{V}}$$

2 $${\rm{Cathode}},{{\rm{O}}}_{2}+2{{\rm{H}}}_{2}{\rm{O}}+4{{\rm{e}}}^{\mbox{--}}\to 4{{\rm{OH}}}^{\mbox{--}},E{^\circ }=+\,0.401\,{\rm{V}}$$

3 $${\rm{Overall}},2{\rm{CO}}{({{\rm{NH}}}_{2})}_{2}+3{{\rm{O}}}_{2}\to 2{{\rm{N}}}_{2}+2{{\rm{CO}}}_{2}+4{{\rm{H}}}_{2}{\rm{O}},E{^\circ }=+\,1.147\,{\rm{V}}$$

At the anode, urea is electro-oxidized to N_2_ and CO_2_ releasing six electrons. The electrons are transferred to the cathode, and consumed in the oxygen reduction reaction generating OH^−^. The OH^−^ is then transported to the anode though the AEM. In the UFC system, the overpotential of urea oxidation reaction (UOR) at anode is considerably higher than that of oxidation reduction reaction (ORR) at cathode, implying that anode catalyst is a key issue for high performance UFCs.

Nickel, an inexpensive metal, catalyzes UOR well, thus it is often used as anode catalyst in UFCs^6,7^. To improve catalytic activity and stability of Ni for UOR, various metals including Co^8^, Cd^9^, Zn^10^, Mo^11^, and Mn^12^ were doped with Ni. Among the different metals to dope with Ni, Co is widely used because it has been reported to reduce the onset potential at which Ni^2+^OH is converted to Ni^3+^OOH, to decrease the catalyst blockage with byproducts, and to suppress unwanted oxygen evolution reaction^8,13,14^.

On the other hand, the catalyst support material also plays a crucial role in improving the catalytic activity of the Ni-based catalysts. The high surface area and good electrical conductivity with high resistance to chemical corrosion are required properties for the efficient supporting matrix^15^. Currently, carbon-based catalysts such as activated carbon, carbon black, carbon nanotubes, carbon nanofibers, graphene oxide, and graphite are most widely used as catalyst supporting material in fuel cells^11,12,15,16^. Three dimensional supports including Ni-foam, carbon (resorcinol- formaldehyde) aerogel, carbon nanotube aerogel, and graphene aerogel are very attractive candidates because of the unique advantages they offer such as low internal resistance to fuel flow, high corrosion resistance, high electrical conductivity, and high surface area for catalyst deposition^16–19^. Especially, carbon nanotube aerogels offer all the above mentioned advantages with the added benefit of a simple preparation process and potential application for different energy storage devices^18,20,21^. For instance, Hameed *et al*.^16^ prepared NiO nanoparticles on different support materials (i.e., activated carbon black, carbon nanotubes, graphene, and graphite), and found that catalyst on graphite showed a better performance for urea oxidation credited to a better diffusion and higher surface area for faster reaction. Furthermore, two and three-dimensional supports for urea electro-oxidation were reported to show superior result compared to those with zero-dimensional and one-dimensional catalyst supports for similar reasons^5,10,22^.

Ni and Co bimetallic nanoparticles decorated, three-dimensional multiwalled carbon nanotube (MWCNT) aerogels, therefore, are expected to offer a unique advantage because it would provide a large surface area for urea adsorption due to its porous structure alongside the decrement of the overpotential by Co doping. In this study, Ni-Co bimetallic nanoparticles decorated MWCNT aerogels with different Co/Ni ratio were prepared using a polyol reduction followed by sol gel method. The structural, morphological, and electrochemical properties of the prepared aerogel catalysts were characterized. The performance of UFCs (urea/O_2_ fuel cells) comprising the composite aerogels as anode materials was evaluated and revealed considerably better performance than other previously reported UFCs.

## Results and Discussion

### Characterization of NiCo/MWCNT-AG

**Figure 1:**
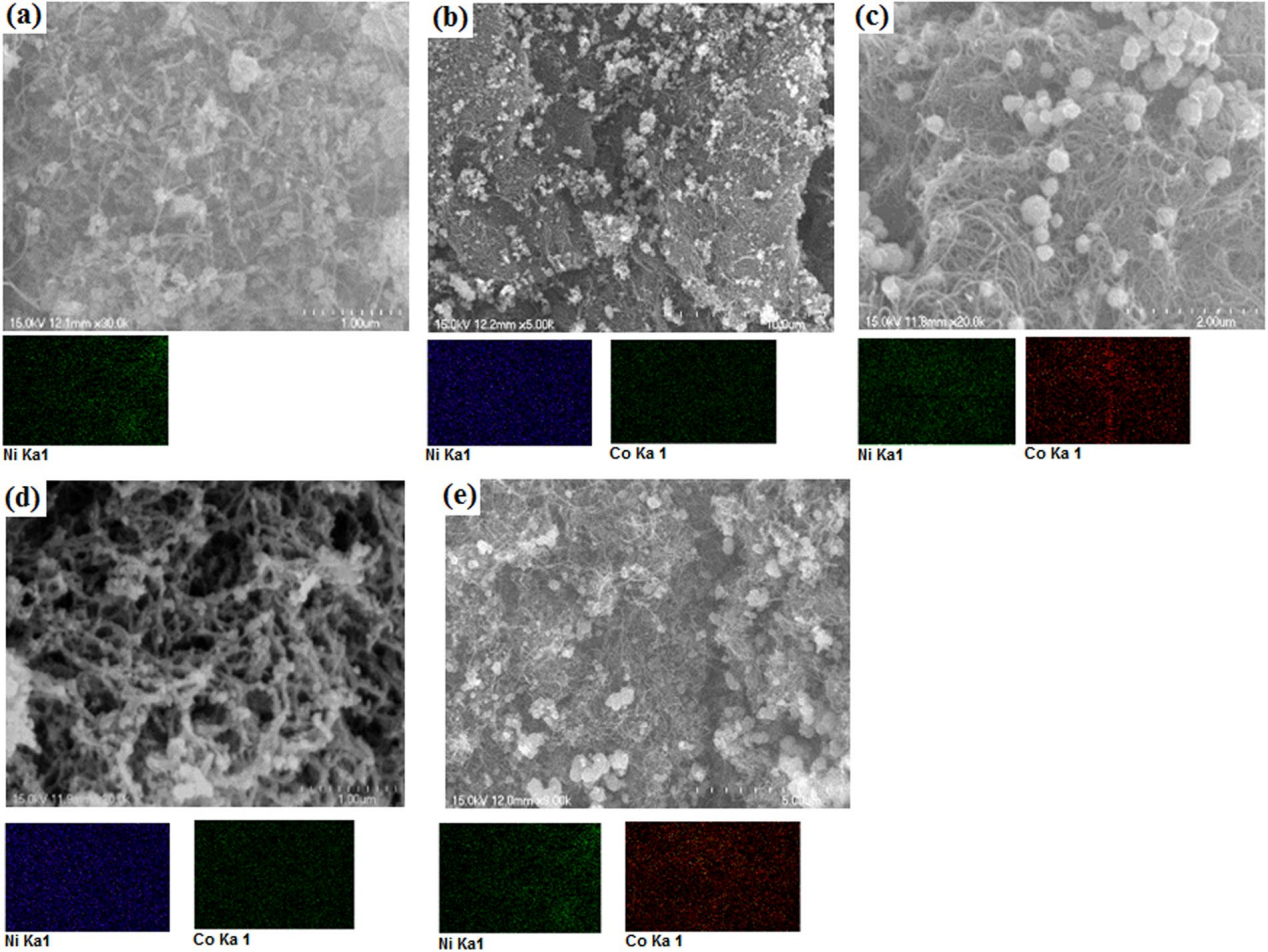
SEM micrographs and elemental mapping of NiCo 0/MWCNT-AG (**a**), NiCo 25/MWCNT-AG (**b**), NiCo 50/MWCNT-AG (**c**), NiCo 100/MWCNT-AG (**d**), and NiCo 200/MWCNT-AG (**e**).

The microstructures of the as prepared aerogels are shown in Fig. 1. As can be seen from the SEM images, a highly interconnected MWCNT networks formed micro, meso and macro pores throughout the structure. The high degree of entanglement that formed a three-dimensional network might be attributed to the crosslinking between hydroxyl group of PVA and hydroxyl and carboxyl group in the outer layer of the MWCNT^23^. In addition, the Ni and Co bimetallic nanoparticles were uniformly distributed throughout the aerogel matrix, mainly due to the good stabilizing and dispersing properties of ethylene glycol^24^. The uniform distribution of Ni/Co was also evidenced by EDX elemental mappings as shown in Fig. 1. The addition of Co, however, affected the size of Ni-Co nanoparticles. The metal particle size decreased with Co contents in the range of 25% (Co/Ni), and then increased as the Co content further increased due to the agglomeration/growth of Co crystals^25^. The average metal particle sizes in NiCo 0/MWCNT-AG, NiCo 25/MWCNT-AG, NiCo 50/MWCNT-AG, NiCo 100/MWCNT-AG, and NiCo 200/MWCNT-AG samples were 60, 40, 100, 150, and 250 nm, respectively. The particle size was measured by an image processing program called Image j^26^; different points from the images were taken to calculate the average size of the nanoparticles of the respective aerogel samples. The metal composition in the aerogel samples, as determined by EDX spectra, was close to those of the theoretical loadings (Supplementary Fig. S1 and Table S1).

The specific surface area of the NiCo/MWCNT-AG samples also varied with Co content. The BET surface area of NiCo 0/MWCNT-AG, NiCo 25/MWCNT-AG, NiCo 50/MWCNT-AG, NiCo 100/MWCNT-AG, and NiCo 200/MWCNT-AG samples were 169, 207, 120, 134, and 34 m^2^ g^−1^, respectively (Suppl. Fig. S2 and Table S2). The result indicated that the NiCo 25/MWCNT-AG sample having the smallest metal particle size exhibited the highest BET surface area. In addition, the average pore size of the NiCo 25/MWCNT-AG sample was the smallest value of 7.9 nm as calculated from the Barrett-Joyner-Halenda (BJH) pore size distribution curve (Supplementary Fig. S2), which was indicative of mesoporosity. Catalyst pore sizes of 2–10 nm range is known to be preferable for electrochemical applications because not only do these pores serve as a good reaction sites, they also shorten the path length the fuel and the ions have to travel^27,28^.

**Figure 2:**
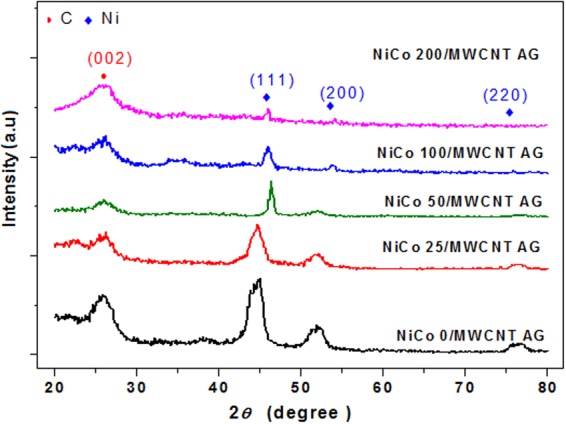
XRD patterns of the different NiCo/MWCNT-AG samples.

Figure 2 shows the XRD patterns of the five aerogel samples. For all samples, peaks at 26° were appeared, which are characteristic peak of carbon in MWCNTs^29^. For the NiCo 0/MWCNT-AG sample, which contained no Co, three diffraction peaks appeared at 44.5°, 51.8°and 76.9° confirming the face centered cubic structure of Ni with indices (111), (200), and (220), respectively (JCPDS No. 04–0850). For the NiCo 25/MWCNT-AG, NiCo 50/MWCNT-AG, NiCo 100/MWCNT-AG, and NiCo 200/MWCNT-AG samples, the peak positions shifted slightly and peak intensity decreased with Co content, indicating that Co affected the crystal structure of the Ni-Co bimetal particles^25^. At higher Co concentrations, peaks gradually disappear, implying the amorphous nature of the Ni-Co bimetal particles.

### Electrochemical characterization of the aerogels

**Figure 3:**
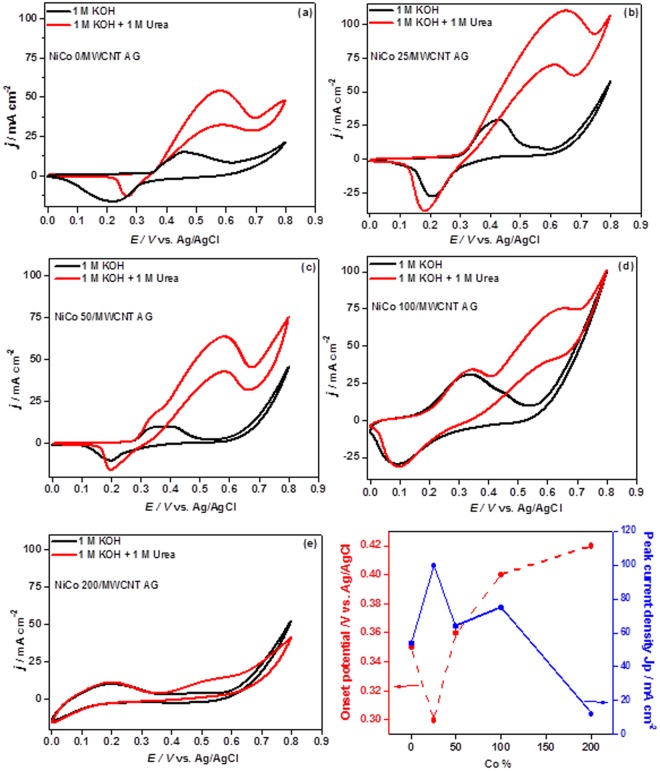
CV curves of NiCo/MWCNT-AGs with different Co contens **(a–e)** at a scan rate of 20 mV s^−1^, and oset potentials and peak current densities for UOR as a function of Co contents **(f)**.

Electrochemical property of NiCo/MWCNT-AG was characterized by cyclic voltammetry (CV) measurements. The CV experiments were run with and without 1 M urea in 1 M KOH in a voltage window between 0 to 0.8 V. Figure 3(a–e) show CV curves of the five different NiCo/MWCNT-AGs containing different Co contents (i.e., NiCo 25/MWCNT-AG, NiCo 50/MWCNT-AG, NiCo 100/MWCNT-AG, and NiCo 200/MWCNT-AG). It can be seen that in the presence of urea all catalysts showed an increase in current density in the forward scan, indicating their activity for urea electroxidation. For NiCo 0/MWCNT-AG (containing no Co) sample, the CV curve (Fig. 3a) in absence of urea showed a pair of redox peaks at 0.45 V and 0.27 V vs. Ag/AgCl corresponding anodic and cathodic peak potentials, respectively, due to the reversible transformation between Ni^2+^(OH)_2_ and Ni^3+^OOH in alkaline medium. In the presence of urea, urea oxidation started at 0.356 V generating a strong current, which was similar to the onset potential of Ni^3+^OOH formation, implying that Ni^3+^OOH catalyzes the urea oxidation reaction (UOR)^30^. In addition, another anodic peak was appeared during the reverse scan at the similar potential for UOR observed in forward scan, implying the regeneration of the active sites for further UOR^30,31^.

By the addition of Co at 25% (Co/Ni), the peak oxidation current density increased considerably and the onset potential of UOR shifted from 0.356 to 0.302, indicating a decrease of 50 mV in the overpotential of UOR. The effect of Co addition on the Ni-based catalysts for UOR might be explained by the enhancement of catalyst surface area by breaking down the Ni-Co bimetallic particles into smaller particles^32,33^ as observed in Fig. 1. The similar results were observed earlier and also explained by the formation of structural defects providing more active sites^14^ and promotion of the electron transfer by allowing Ni to reach a higher oxidation state^34^. However, further increase of Co contents above 25% pushed the onset potential to the positive direction and resulted in a significant decrease of the urea oxidation current. This result might be because the Co, which is in active for UOR, reduced the exposed Ni active site^13,14^. In addition, it was observed that the size of Ni-Co bimetallic particles increased with the decrease in BET surface area as Co contents increased above 25%. Figure 3f shows the plot of onset potential and peak current density for UOR as a function of Co contents. The lowest onset potential and the highest peak current density for UOR were obtained at 25% of Co content. Therefore, NiCo 25/MWCNT-AG was chosen as the best catalyst and further electrochemical experiments were done on it.

**Figure 4:**
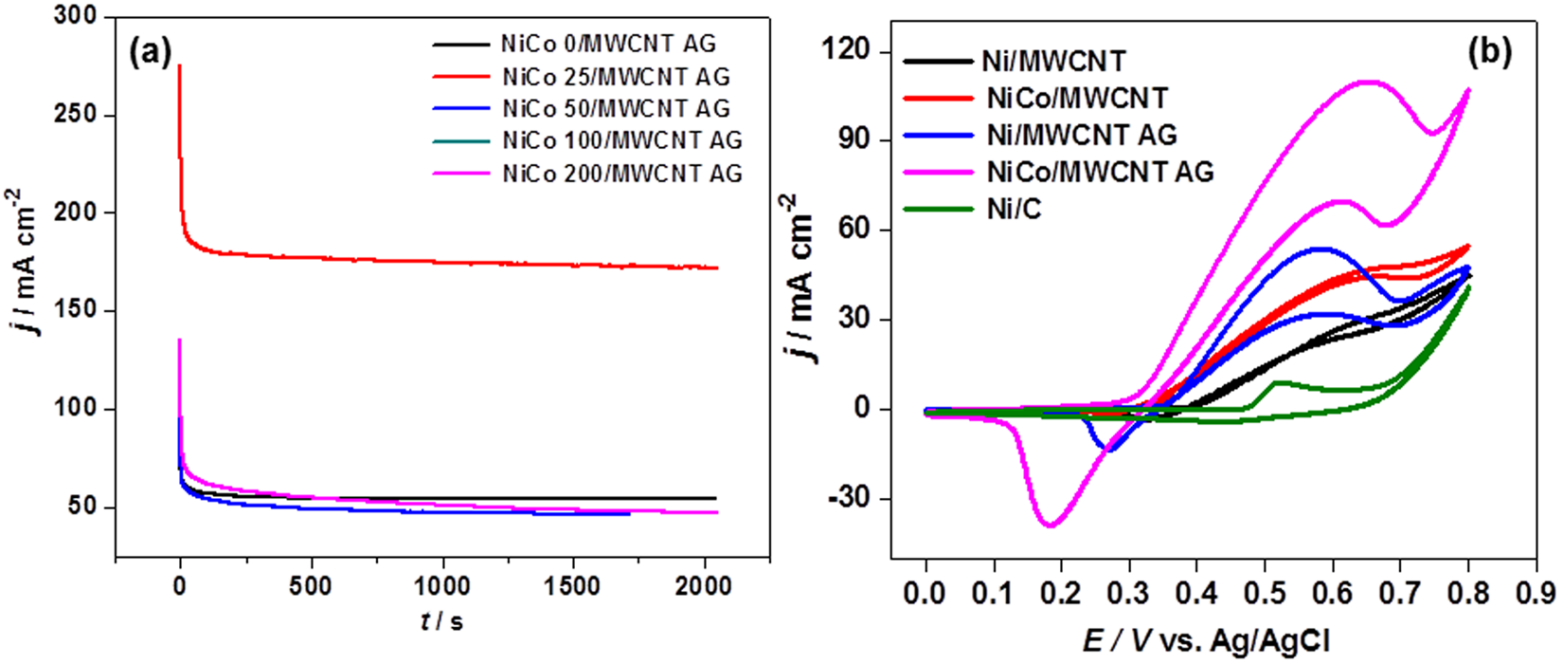
Chronoamperometric measurment of the five aerogel catlysts at 0.7 V (vs. Ag/AgCl) in 1 M urea in 5 M KOH (**a**) and CV curves of the various catalysts with different supporting materials in the presence of 1 M urea in 1 M KOH at the scan rate 20 mV s^−1^ (**b**).

The electro-catalytic activities of NiCo/MWCNT-AG catalysts were further examined by chronoamperometric measurements at 0.7 V in 1 M urea in 5 M KOH, as shown in Fig. 4a. All the aerogel electrodes exhibited stable current responses. However, NiCo 25/MWCNT-AG showed much higher current density as also observed in the above CV measurements, suggesting a potentially high constant power output when used as anode catalyst.

To examine the effect of MWCNT aerogel as a supporting material on the activity of the Ni-Co bimetallic catalyst for UOR, the Ni-Co bimetallic particles was incorporated into MWCNT powder and compared their CV curves. Figure 4b shows the CV curves of Ni/MWCNT, NiCo 25/MWCNT, Ni/MWCNT-AG, NiCo 25/MWCNT-AG, and Ni/C. Ni/C, which is a commercial Ni catalyst on carbon black, was included for the sake of comparison. Ni/MWCNT-AG exhibited significantly a lower onset potential with a higher peak current for UOR than Ni/MWCNT and Ni/C. In addition, NiCo/MWCNT-AG also exhibited significantly a lower onset potential with a higher peak current than NiCo/MWCNT. The result, therefore, implied that MWCNT aerogel structure provided a high surface area and allowed fast diffusion of urea and products to facilitate the UOR. Electrochemically active surface area (EASA) was calculated from the CV data as described elsewhere^35^. The calculation procedure and other electrochemical parameters extracted from the CV data are presented in Supplementary Table S2. The EASAs of Ni/MWCNT, NiCo/MWCNT, Ni/MWCNT-AG, NiCo/MWCNT-AG, and Ni/C were 3.0, 4.7, 15.3, 78.5, and 0.001 m^2^ g^−1^, respectively. The highest EASA of NiCo/MWCNT-AG was ascribed to the Co effect and a porous 3D network structure of MWCNT aerogel.

### Effect of scan rate

**Figure 5:**
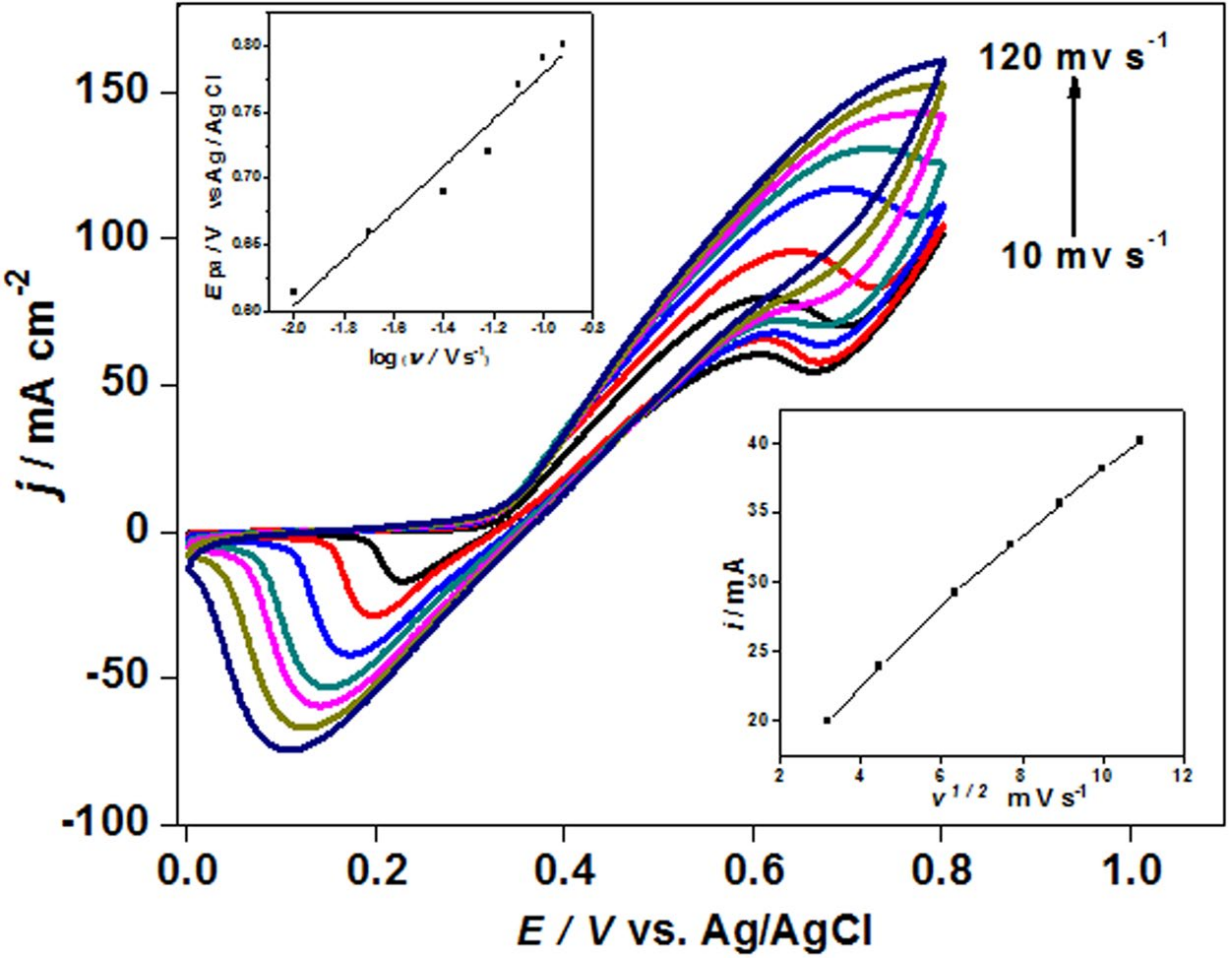
CV curves of NiCo 25/MWCNT-AG in 1 M urea in 1 M KOH at different scan rates, insets are the anodic peak currents vs. the square root of scan rate (bottom) and anodic peak potentials vs. the logarithm of scan rate (top).

The electrochemical reaction mechanism of NiCo 25/MWCNT-AG catalyst towards UOR was studied by examining the effect of scan rate on the peak potential and current density. Figure 5 shows CV curves in the presence of 1 M urea in 1 M KOH at different scan rates ranging from 10 to 120 mV s^−1^. As shown in inset of Fig. 5, anodic peak current increased linearly with the square root of the scan rate, suggesting that the electrochemical reaction on the electrode surface was a diffusion-controlled process according to the Randles-Sevcik model^36^. Furthermore, the anodic peak potential (*E*_pa_) increased linearly with the logarithm of the scan rate, impling kinetic limitations on the UOR^30^. From these results, it was concluded that urea electro-oxidation on NiCo 25/MWCNT-AG electrode was mixed control reaction, as observed previously^5,8,13,14^.

**Figure 6:**
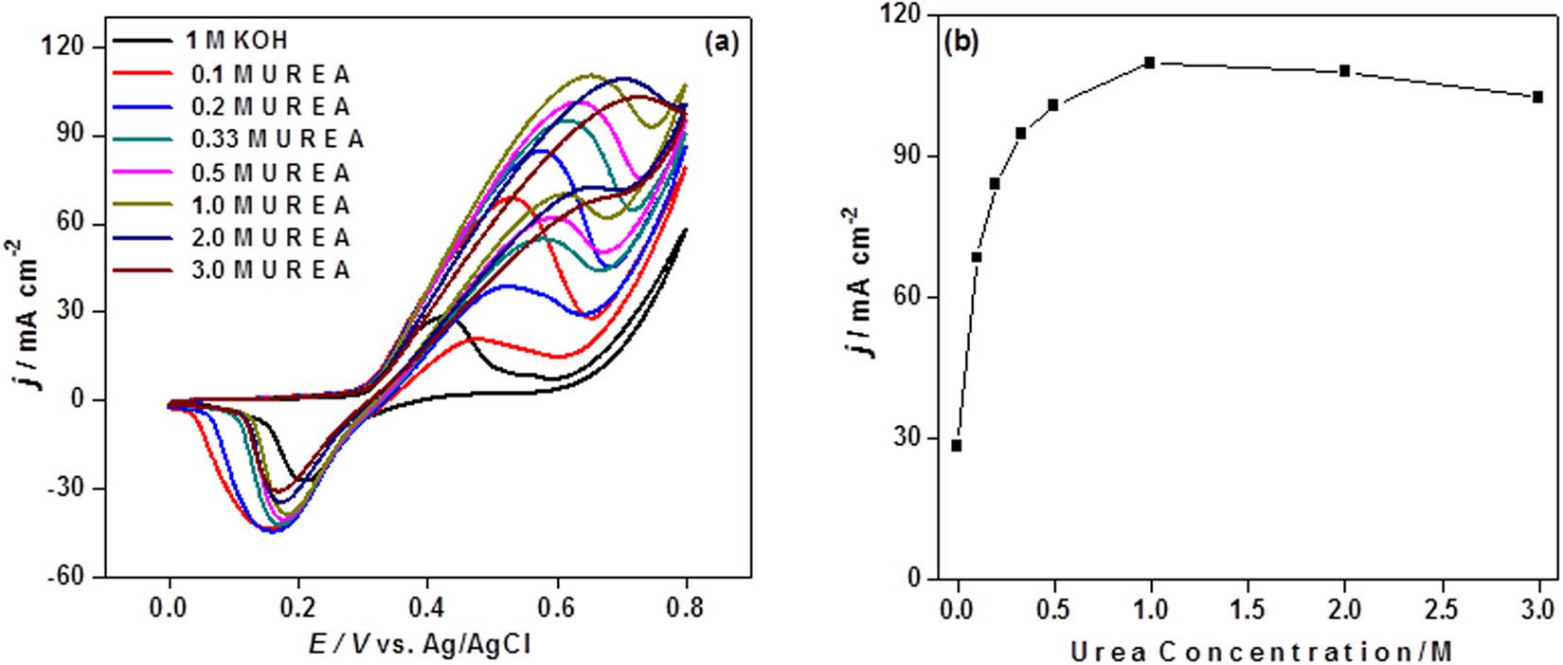
CV curves of NiCo 25/MWCNT/AG at different urea concentration in 1 M KOH at a scan rate of 20 mV s^−1^ (**a**) and the effect of urea concentration on peak current density (**b**).

From the plot of the urea oxidation peak potential vs. scan rate (Inset of Fig. 6, bottom), the diffusion coefficient of urea molecules (D) was estimated according to the following equation^30^:

4 $${I}_{pa}=(2.99\times {10}^{5})n{[(1-\alpha ){n}_{o}]}^{1/2}{{\rm{ACD}}}^{1/2}{v}^{1/2}$$

where *I*_pa_ is urea oxidation peak current, *n* (6) is the total number of electrons involved in UOR, α is the anodic transfer coefficient, *n*_o_ (1) is the number of electrons involved in rate determining step. A (0.25 cm^2^) is the electrode surface area, C (1 M) is the urea concentration, and *v* is the scan rate. The following equation was used to calculate *α*^30^:

5 $$E{\rm{pa}}=[0.03/(\alpha {n}_{o})]\,\mathrm{log}\,v+{\rm{constant}}$$

The diffusion coefficient of urea in the NiCo 25/MWCNT-AG electrode was then calculated to be 0.451 × 10^−5^ cm^2^s^−1^, which is considerably higher than reported values for activated carbon, MWCNT, graphene, and graphite supports^16^, mainly because of a porous 3D network structure of MWCNT aerogel.

### Effect of urea and KOH concentration

To choose the optimum anolyte (urea) concentration in a urea/O_2_ fuel cell, CV experiments were carried out with different concentration (0.1–3 M) of urea in 1 M KOH, as shown in Fig. 6. The urea oxidation peak current density increased as the urea concentration increased up to 1 M. However, at high urea concentration above 1 M, the current leveled off and decreased slightly, indicating a saturation kinetics of UOR on the NiCo 25/MWCNT*-*AG. Furthermore, the urea oxidation peak potential oxidation also increased with the urea concentration, implying a higher overpotential at a high urea concentration. At a high urea concentration, the local surface coverage of the Ni catalyst by excess urea and reaction products might restrict the contact with OH^−^. The OH^−^ is required for the formation of NiOOH, which is the active catalyst for UOR. Therefore, this result indicated that an optimization of urea concentration is required to obtain a high power density by considering current output and overpotential, simultaneously.

**Figure 7:**
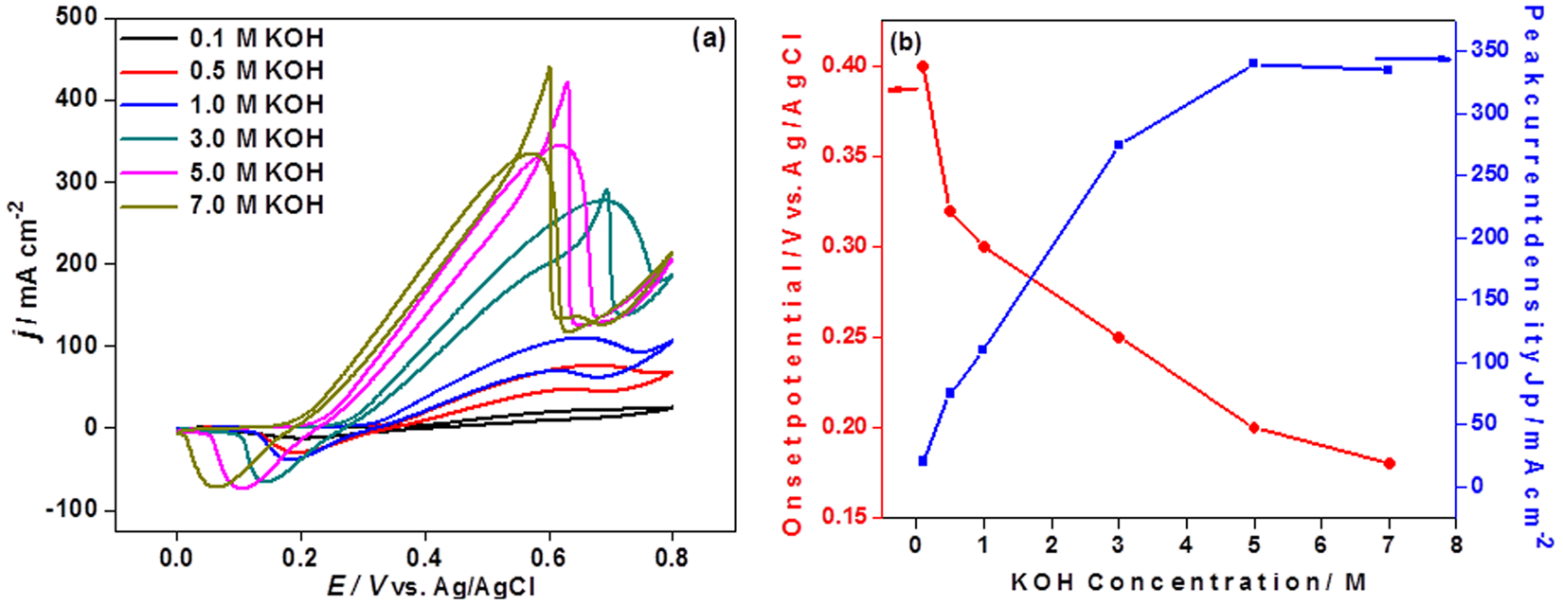
CV curves of NiCo 25/MWCNT/AG at different KOH concentration at a scan rate of 20 mV s^−1^ (**a**) and the effect of KOH concentration on peak current density at 1 M urea (**b**).

Figure 7 shows CV curves of NiCo 25/MWCNT-AG in the presence of 1 M urea in KOH of different concentrations (1–7 M). The onset potentials for urea oxidation decreased as KOH concentration increased within the scope of the experiment as expected because OH^−^ is the reactant for the formation of active catalyst, NiOOH. On the other hand, the urea oxidation peak current increased with KOH concentration up to 5 M, and then remained constant, probably because at high KOH concentration, the viscosity of urea solution increased and thus the diffusion of urea molecules was retarded. Furthermore, a high KOH concentration might lead to possible crossover, catalyst oxidation, oxygen evolution reaction, and accumulation of unwanted byproducts such as potassium carbonate. Therefore, the appropriate KOH concentration to go along with 1 M urea was appeared to be 5 M.

### Urea/O_2_ cell tests

**Figure 8:**
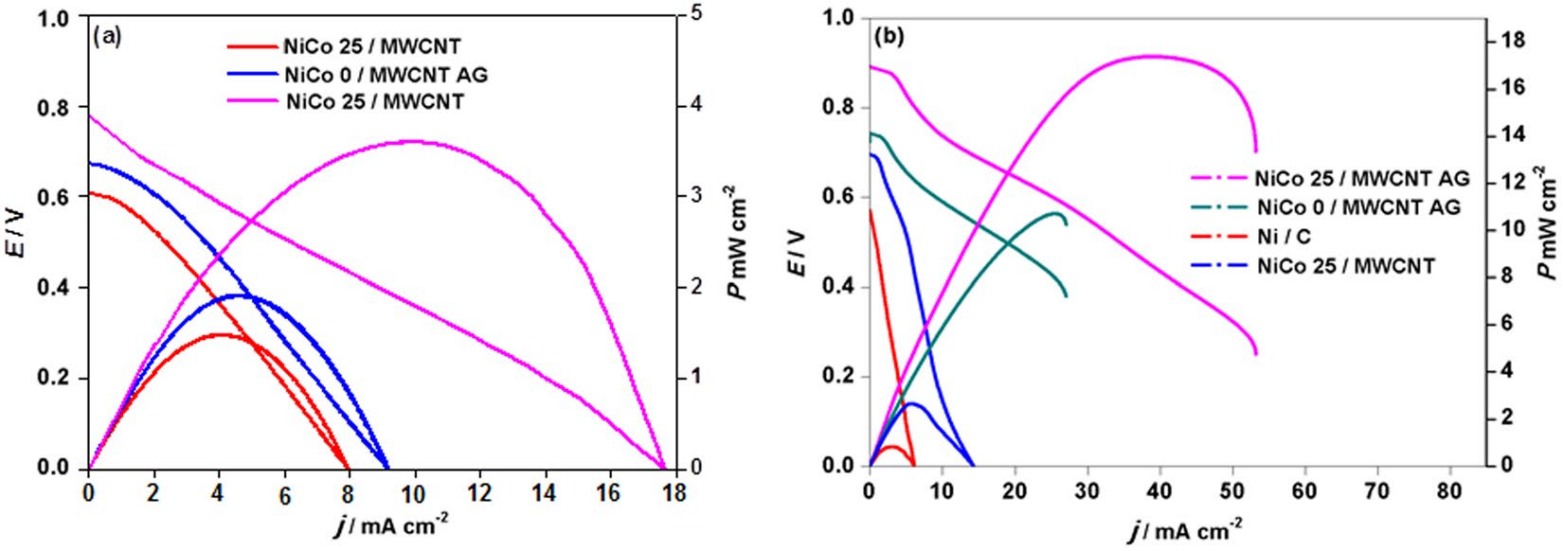
Performance of urea/O_2_ fuel cells with different anode materials (NiCo 25/MWCNT, NiCo/MWCNT-AG, NiCo 25/MWCNT-AG, and Ni/C) using 1 M urea in 3 M KOH as fuel and humidification oxygen as oxidant at 20 °C (**a**) and 60 °C (**b**).

The performances of NiCo 25/MWCNT, NiCo/MWCNT-AG, NiCo 25/MWCNT-AG, and Ni/C as anode in a urea/O_2_ fuel cell were evaluated. Figure 8 shows the I–V polarization and power density curves of those cells recorded in 1 M urea in 3 M KOH as anolyte. It should be noted that although the optimum KOH concentration was found to be 5 M in CV tests, it was observed in fuel cell tests that at 5 M KOH, a transition zone (i.e., decrease of current density) was observed in the polarization curve, indicating catalyst site blockage^22^. The maximum power densities of cells compring NiCo 25/MWCNT, NiCo/MWCNT-AG, NiCo 25/MWCNT-AG, and Ni/C anode catalysts were 2.7, 10.7, 17.5, and 0.8 mWcm^−2^ at 60 °C, respectively, in agreement with their CV results. The best fuel cell performance was observed for the NiCo 25/MWCNT-AG anode catalyst, which was mainly ascribed to its higher BET surface area and EASA, as observed earlier. By comparing the performances of NiCo 0/MWCNT-AG and NiCo 25/MWCNT-AG, it is clearly shown that Co-doping significantly enhanced the maximum power density, because appropriate amount of Co doping decreased the overpotential of UOR as observed earlier. In addition, NiCo 25/MWCNT-AG exhibited considerably better performance than NiCo 25/MWCNT, indicating the catalyst supporting matrix were of critical and the highly porous 3D MWCNT aerogel provided a higher surface area and faster for mass transport for the UOR. The performance of urea/O_2_ fuel cells was also significantly affected by operating temperature; the maximum power density increased 2–5 times as the temperature increased from 20 to 60 °C (Fig. 8a,b), mainly because of the concomitant increase of electrochemical redox reaction rates and the ion conductivity of the AEM^37,38^.

## Conclusion

Ni-Co bimetallic nanoparticles decorated MWCNT aerogels were synthesized by simple polyol reduction and sol-gel method. As prepared Ni-Co/MWCNT aerogels exhibited a highly porous 3D network structures with uniform distribution of Ni-Co nanoparticles. The appropriate amount of Co-doping significantly increased peak oxidation current and reduced the overpotential of UOR. Furthermore, the MWCNT aerogel also remarkably enhanced electro-catalytic activity of the Ni-Co bimetallic catalysts by providing a high surface area and fast mass transport for the UOR. A urea/O_2_ fuel cell containing Ni-Co/MWCNT aerogels as an anode catalyst achieved a maximum power density of 17.5 mW cm^−2^ with 1 M urea in 3 M KOH as a fuel at 60 °C, outperforming previously reported UFCs. The result suggested that the Ni-Co/MWCNT aerogels are promising anode materials for urea fuel cells.

## Methods

### Synthesis of Ni-Co decorated MWCNT aerogels

The MWCNTs were functionalized by being refluxed in a mixture of concentrated H_2_SO_4_ and HNO_3_ (3:1 by volume) for 3 h^39^. The mixture was then filtered and washed with de-ionized (DI) water repeatedly. The filter cake was dried overnight in vacuum oven at 80 °C. Ni and Co binary metal nanoparticles were deposited onto the functionalized MWCNT by a simple polyol-reduction method^40^. Briefly, CoCl_2_ and NiCl_2_ were dissolved in 200 mL of ethylene glycol at 0.02 M of the total metal concentration. Afterwards, 500 mg of MWCNT was added and sonicated for 10 min to insure uniform dispersion. Then, to the above mixture, 5 mL of NaOH (1 M in ethylene glycol) was added and followed by the addition of 2.4 mL of hydrazine under vigorous stirring. The reaction was kept at 80 °C for 3 h. The solution was filtered, and the filter cake was washed with acetone, ethanol, and DI water multiple times, and stored in a closed container overnight. The filter cake was then freeze dried for 48 h, followed by drying in vacuum oven at 50 °C for 6 h to obtain Ni-Co bimetallic nanoparticles decorated MWCNT (Ni-Co/MWCNT) powder.

The Ni-Co/MWCNT hydrogels were prepared by a sol-gel method. In a glass vial, 300 mg of NiCo/MWCNT powder was mixed with 20 mL of polyvinyl alcohol (PVA) aqueous solution (1 wt%) and 5 mL of acetic acid under mild stirring. Then, 750 μL of glutaraldehyde (25 wt%) was added and sonicated for 10 min followed by stirring for 30 min. Afterwards, the vial was heated at 90 °C in an oil bath for 12 h to obtain NiCo/MWCNT/PVA hydrogels. The hydrogels were carefully washed with DI water several times and kept in a refrigerator at −20 °C overnight. The frozen hydrogels were then freeze dried at −78 °C for two days to obtain Ni-Co/MWCNT/PVA aerogels. Five samples were prepared which had different Co/Ni ratios, and denoted as NiCo/MWCNT-AG. The composition of the NiCo/MWCNT-AG is summarized in Supplementary Table S1.

### Preparation of electrodes and urea/O_2_ fuel cell testing

To prepare catalyst inks, as synthesized catalyst powders (NiCo/MWCNT and NiCo/MWCNT-AG) were dispersed in dimethylformamide and mixed with Nafion solution (5 wt % in isopropanol). After homogenization, the resulting anode catalyst ink was coated onto a carbon paper (TGP-H-60 Toray) to achieve a catalyst loading of 2 mg cm^−2^. Similarly, a commercial Pt/C (40 wt%, E-TEK)-coated carbon paper with a Pt loading of 2 mg cm^−2^ was used as a cathode. An anion exchange polymer membrane (AEM, Fumasep FAB-PK-130) was used as a polymer electrolyte. The AEM was immersed in 1 M KOH solution and heated at 50 °C for 2 h, and then left in the solution for 10 h as a pretreatment. After washing with DI water repeatedly, the membrane was sandwiched between the anode and cathode, and was hot pressed at 120 °C for 1 min to obtain membrane electrode assembly (MEA). The unit cell possessed an active area of 5.0 cm^2^. A graphitic plate with serpentine flow channels and gold-coated stainless current collector were used for the single cell test. Aqueous solution of 1 M urea in 3 M KOH was fed into the anode side at a rate of 3 mL min^−1^, and humidified oxygen was supplied to the cathode a rate of 200 sccm.

### Analysis

The aerogel samples were hand crushed using a mortar and pestle into powder for the analysis. The crystal structure of the sample powder was analyzed by X-ray diffractometer (XRD, Rigaku) with Cu Kα radiation (λ = 1.5418 Å) at 2 *θ* between 20 to 90^o^ with the accelerating voltage of 40 keV and a current of 30 mA at a scan rate of 2 °/min. The morphology and composition of the samples were analyzed by a scanning electron microscope (SEM, A JEOL JSM-6700F) equiped with an energy dispersive X-ray spectroscope (EDX). The specific surface area was also measured along with pore size distribution using Brunauer-Emmett-Teller (BET) and Barrett-Joyner-Halenda (BJH) methods, respectively.

The electrochemical measurements were done by cyclic voltammetry (CV) using a potentiostat-galvanostat (VSP, Bio-Logic) on conventional three electrode cells set up. Ag/AgCl electrode was used as a reference electrode and a Pt wire as a counter electrode. Catalyst powder applied on carbon paper (as described above) cut into 5 mm $\times$ 5 mm was used as working electrode. Fuel cell performance was evaluated utilizing the potentiostat-galvanostat interfaced with EC-lab 11.01 data acquisition software.

## Supplementary information

Supplementary Information

## Data Availability

The datasets generated during and/or analysed during the current study are available from the corresponding author on reasonable request.
