# Supplementary material for: Ni-Co bimetal decorated carbon nanotube aerogel as an efficient anode catalyst in urea fuel cells
Source: Sci Rep. 2019 Jan 24;9:479. doi: 10.1038/s41598-018-37011-w (PMC6345754; doi:10.1038/s41598-018-37011-w)
Supplement: Supplementary file 1 — Supplementary Information [file 41598_2018_37011_MOESM1_ESM.docx]

***Electronic Supplementary Information for:***

**Ni-Co bimetal decorated carbon nanotube aerogel as an efficient anode catalyst in urea fuel cells**

Robel Mehari Tesfaye, Gautam Das, Bang Ju Park, Jihyeon Kim, and Hyon Hee Yoon*

**Fig. S1.** EDX spectra of (a) NiCo 0/MWCNT-AG, (b) NiCo 25/MWCNT-AG, (c) Ni-Co 50/MWCNT-AG, (d) NiCo 100/MWCNT- AG, and (e) NiCo 200/MWCNT-AG.


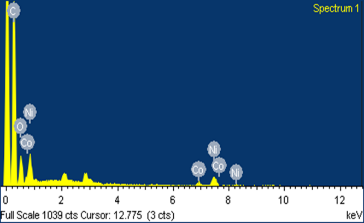

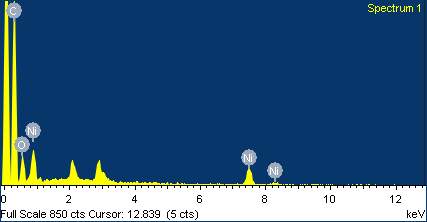


**(b)**

**(a)**


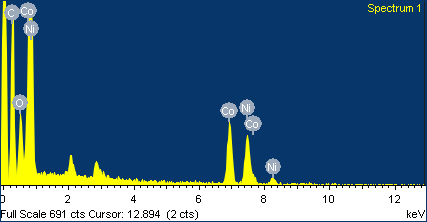

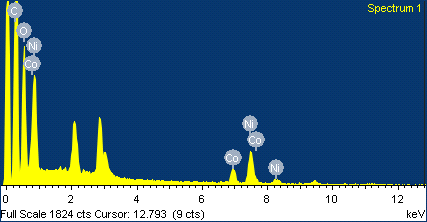


**(c)**

**(d)**


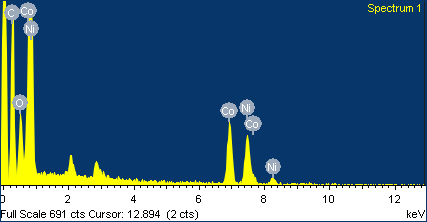


**(e)**

**Table S.1.** Ni/Co ratios in Ni-Co/MWCNT-AG samples as determined by EDX analysis.

| Samples | Ni:Co ratio in  precursor solution | | Ni:Co ratio as  measured by EDX | |
| --- | --- | --- | --- | --- |
| NiCo 0/MWCNT-AG | | 1 : 0 | | 100 : 0 |
| NiCo 25/MWCNT-AG | | 4 : 1 | | 3.72 : 1 |
| NiCo 50/MWCNT-AG | | 2 : 1 | | 2.00 : 1 |
| NiCo 100/MWCNT-AG | | 1 : 1 | | 1.02 : 1 |
| NiCo 200/MWCNT-AG | | 1 : 2 | | 1 : 1.72 |

**Fig S2**. N_2_ adsorption isotherms and the pore diameter distribution of Ni-Co/MWCNT-AG samples.


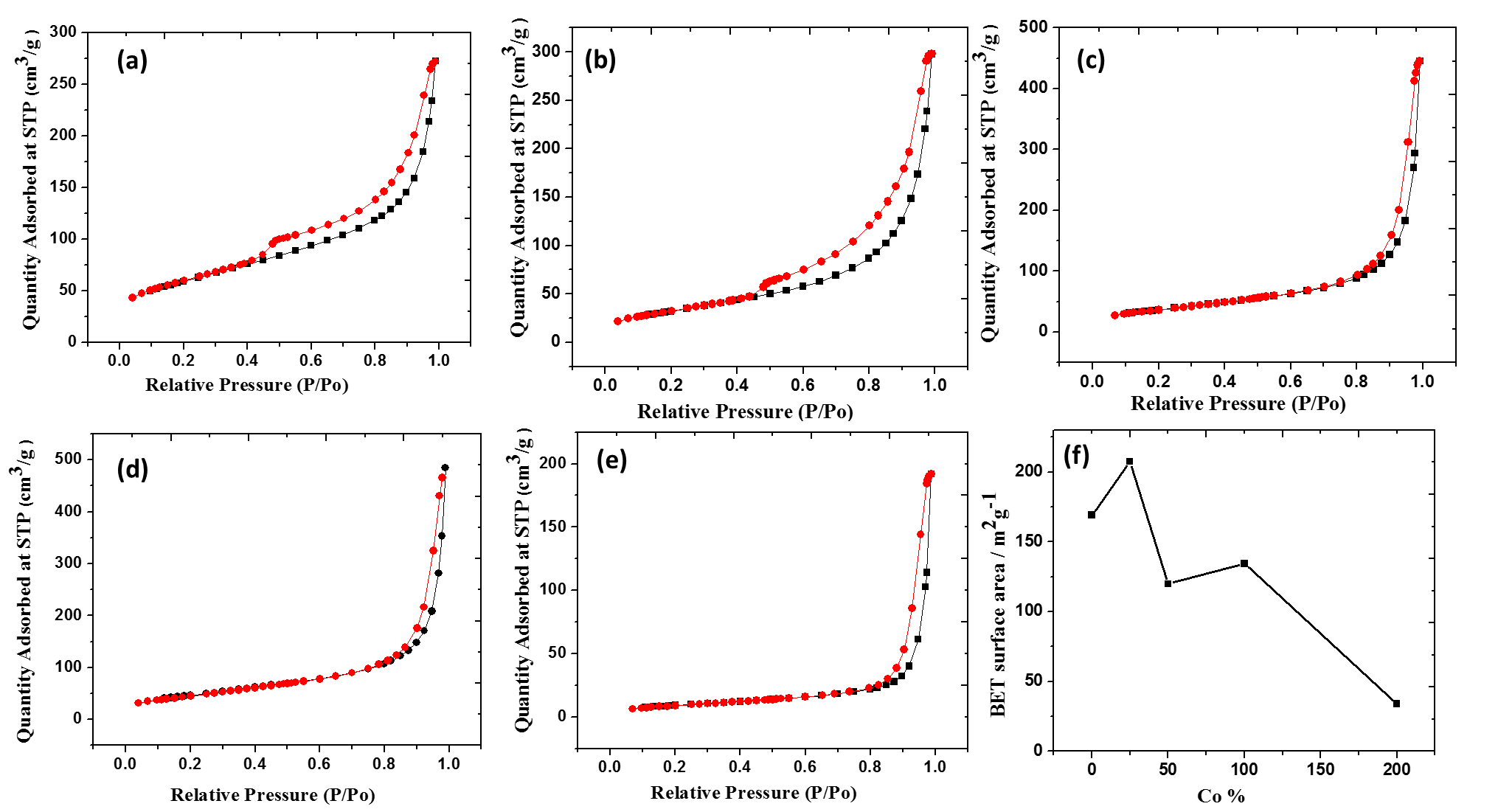

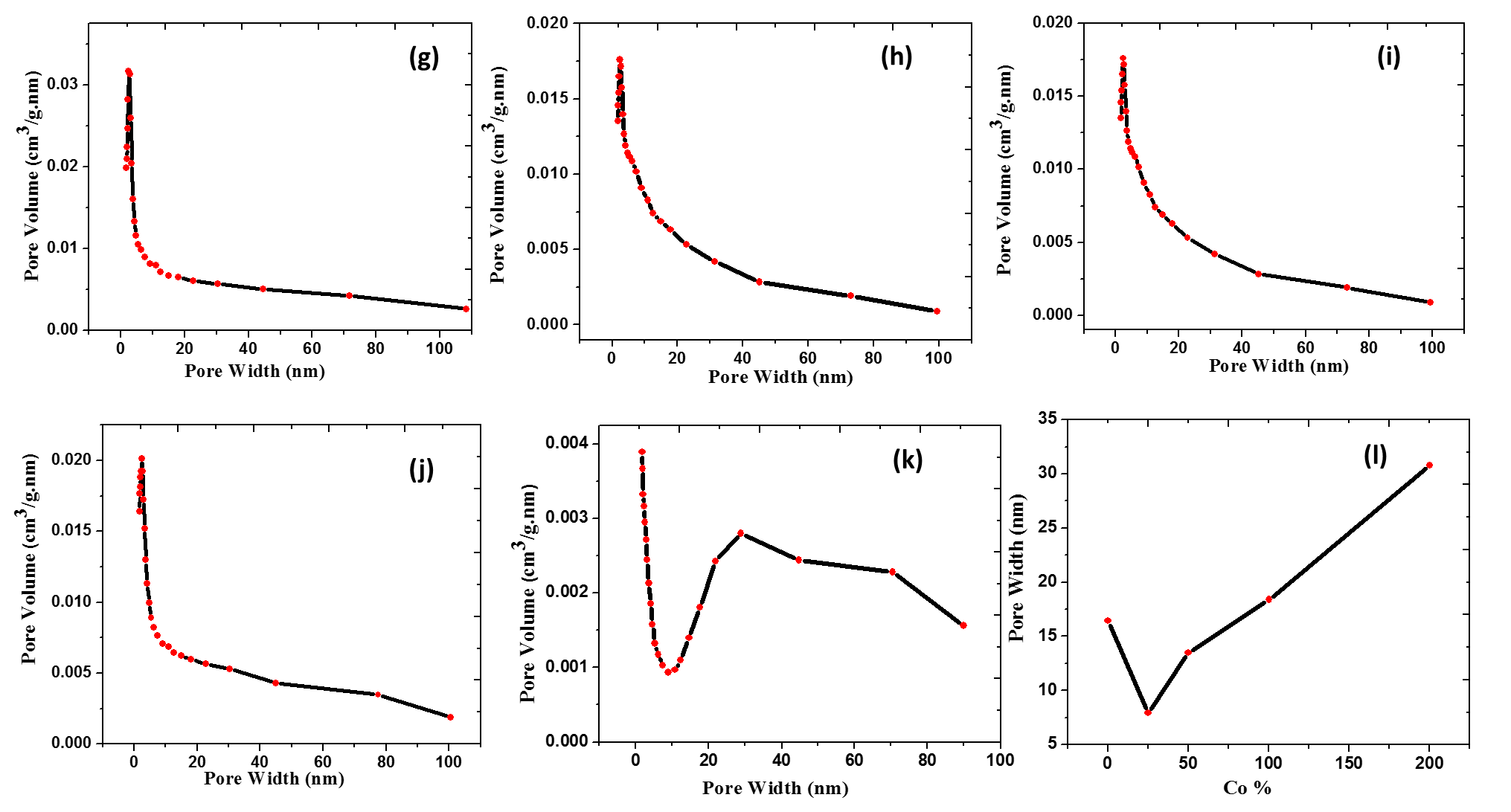


| Sample  electrodes | Q,  mA V | EASA,  m^2^g^-1^ | Mass activity,  mA mg^-1^ | Specific activity,  mA cm^-2^ mg^-1^ |
| --- | --- | --- | --- | --- |
| Ni/C | 0.01095 | 0.0013 | 176.46 | 176.46 |
| Ni/MWCNT | 0.03983 | 3.0393 | 294.11 | 294.11 |
| NiCo/MWCNT | 0.06111 | 4.6621 | 441.70 | 441.70 |
| Ni/MWCNT-AG | 0.20014 | 15.270 | 529.41 | 529.41 |
| NiCo/MWCNT-AG | 1.02909 | 78.514 | 1078.4 | 1078.4 |

**Table S2.** Electrochemically active surface areas (EASAs) of different UOR catalysts.

a) The ECSA was estimated according to the following equation:

ECSA = $\frac{Q}{m q}$

where Q is the charge required to reduce Ni^3+^ to Ni^2+^, *m* is the loading of Ni, and *q* is 257 μC cm^−2^ for the one electron process in the conversion of NiOOH to Ni(OH)_2_. *Q* was calculated by integrating the peak from the CV curves.

b) Mass activity was calculated by dividing the anodic peak current by the Ni-Co loading.

c) Specific activity was estimated by dividing the mass activity by the ESCA.

(Here, geometric area of the electrodes was 0.25 cm^2^, the catalyst loading (Ni+Co) was 0.255 mg.)
